# Validation of inflationary non-invasive blood pressure monitoring in emergency room patients

**DOI:** 10.1186/cc13310

**Published:** 2014-03-17

**Authors:** J Sasaki, S Hori

**Affiliations:** 1 Keio University School of Medicine, Tokyo, Japan

## Introduction

Currently, most non-invasive blood pressure (NIBP) monitoring is based on the oscillometric method and determines the blood pressure during cuff deflation [1]. On the other hand, a measurement during cuff inflation may be advantageous, as cuff inflation requires lower cuff pressure and shorter duration than deflation. In surgical patients during anesthesia, the inflationary NIBP has reasonable accuracy compared with conventional deflationary NIBP [2]. Few studies have reported NIBP monitoring using the inflationary NIBP in ER patients with various unstable conditions. A purpose of this study is to verify the usefulness of the inflationary NIBP monitoring in the emergency department.

## Methods

A total of 2,981 NIBP data were collected from 174 patients (age; 56.5 ± 22.2 (7 to 92) years) who have been accommodated in the resuscitation area of the ER at Keio University Hospital, using alternately two algorithms with a standard monitor (BSM-6000; Nihon Kohden Inc., Tokyo, Japan). One algorithm consists of continuous inflationary and deflationary measurement in a single cycle (dual algorithm, 1,502 data) performed in order to verify a success rate and a precision of data. The deflationary algorithm (1,479 data) consists of only conventional deflationary measurement performed in order to verify the duration of the measurement cycle.

## Results

**Figure 1:**
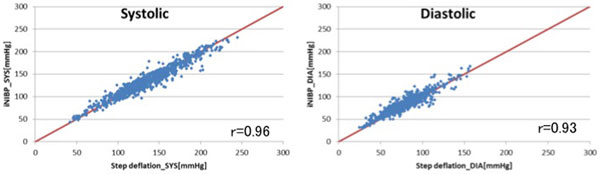
**Precision of measurements between inflationary and deflationary NIBP**.

**Figure 2:**
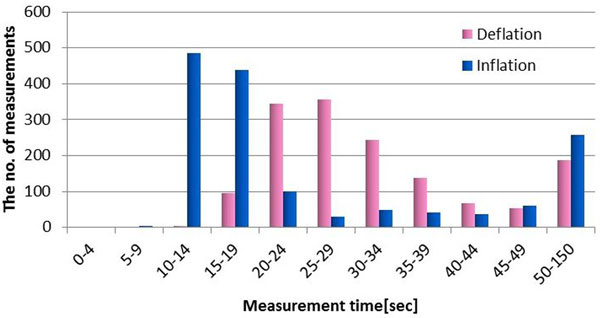
**Frequency histogram of measurement time**.

The success rate of the inflationary NIBP (completed only by inflationary method) was 69.0%. The bias and precision of systolic pressure and diastolic pressure (difference of systolic and diastolic pressure between inflationary and deflationary NIBP) were -0.6 ± 8.8 and 3.5 ± 7.5 mmHg, respectively (Figure 1). Inflationary NIBP could also determine NIBP more quickly compared with deflationary NIBP (16.8 vs. 29.1 seconds, median) (Figure 2).

## Conclusion

These data suggest that inflationary NIBP has reasonable accuracy and sufficient rapidity compared with deflationary NIBP in emergency room patients.

## References

[B1] van Montfrans GA Blood Press Monit 2001 6 287 290 10.1097/00126097-200112000-00004 12055403

[B2] Onodera J JAnesth 2011 25 127 13021188429

